# On the 3D point clouds–palm and coconut trees data set extraction and their usages

**DOI:** 10.1186/s13104-023-06647-x

**Published:** 2023-12-08

**Authors:** Chantana Chantrapornchai, Phisit Srijan

**Affiliations:** Department of Computer Engineering, Kasetsart University, Ngamwongwan Rd., Bangkok, 10900 Thailand

**Keywords:** Palm data set, Coconut dataset, Drone image analytic, WebODM, Open3D, CloudCompare

## Abstract

**Objective:**

Drone image data set can be utilized for field surveying and image data collection which can be useful for analytics. With the current drone mapping software, useful 3D object reconstruction is possible. This research aims to learn the 3D data set construction process for trees with open-source software along with their usage. Thus, we research the tools used for 3D data set construction, especially in the agriculture field. Due to the growing open-source community, we demonstrate the case study of our palm and coconut data sets against the open-source ones.

**Results:**

The methodology for achieving the point cloud data set was based on the tools: OpenDroneMap, CloudCompare, and Open3D. As a result, 40 palm trees and 40 coconut tree point clouds were extracted. Examples of the usages are provided in the area of volume estimation and graph analytics.

## Introduction

In agriculture, field surveying using drones is a common method to collect data. Drone images are used to analyze plant growth and crop yield. The collected data are stitched into 2D orthomosaic images. Combining other drone data with the georeference points, more information can be obtained such as height. This information can be used to construct 3D field models.

Constructing a 3D data set requires effort and involves many software tools. In the agriculture field, after drone flying, software is needed to perform orthomosaic and 3D construction. Current options available are divided into both commercial and non-commercial. Previous work in [1] compares orthomosaic and photogrammetry software. In the article, most mentioned ones are commercial such as DroneDeploy [2], Pix4D Mapper [3], AutoDesk$^{\textrm{R}}$ Recap [4], 3DF [5], Agisoft PhotoScan [6], while the open-source one is OpenDroneMap (ODM) [7].

For example, DroneDeploy is a platform with both enterprise and individual licenses available [2]. As of 2023, plans start at 329 USD per month, allowing for up to 3K images per map. This includes services such as orthophoto, plant health, and GCP. Pix4D Mapper focuses on photogrammetry tasks. It creates 3D maps from 2D maps by constructing surfaces, volumes, and cloud points. The minimum monthly subscription for Pix4D Mapper is 291 USD, with a floating license available for 4,900 USD [3] (as of 2023) Agisoft PhotoScan is another one that focuses on photogrammetry which includes the feature of detecting powerlines. Three pricing models are node-lock license, floating license, and educational license [6]. The basic edition of Agisoft PhotoScan offers features such as photogrammetric triangulation, dense point cloud generation and editing, 3D models generation and texturing, diffuse, occlusion, and normal texture map generation,etc. Undoubtedly, the software features are excellent, while pricing model may be unaffordable for beginners. Therefore, the open-source version is one of the solutions, as it can be deployed at no cost and further customized to specific needs.

In [8], 6 free drone mapper software were mentioned. Among these are DJI GS Pro and Pix4Dcapture which provide the flight planning feature. SkyeBrowse and DroneDeploy offer limited days for trial use. OpenDroneMap [7] is the option with the source code in github containing more than 2K stars, which is the target for our research.

Our research aims to study the process of 3D point clouds and their feature extraction using open-source drone mapping software. After the 3D data set is constructed, there can be many analytics applications upon it. WebODM [9] is the main selected tool for orthoimages and 3D point cloud constructions. The data set collected from palm and coconut field surveys in Thailand is the case study.

## Methods

### Data source and overall data processing

Our initial data set for the study was collected from the drone survey in 2022. The area size is 345,686.94 m$^2$ and 224,573 m$^2$ respectively, for palm and coconut fields, in Pathum Thani province in Thailand.

**Fig. 1:**
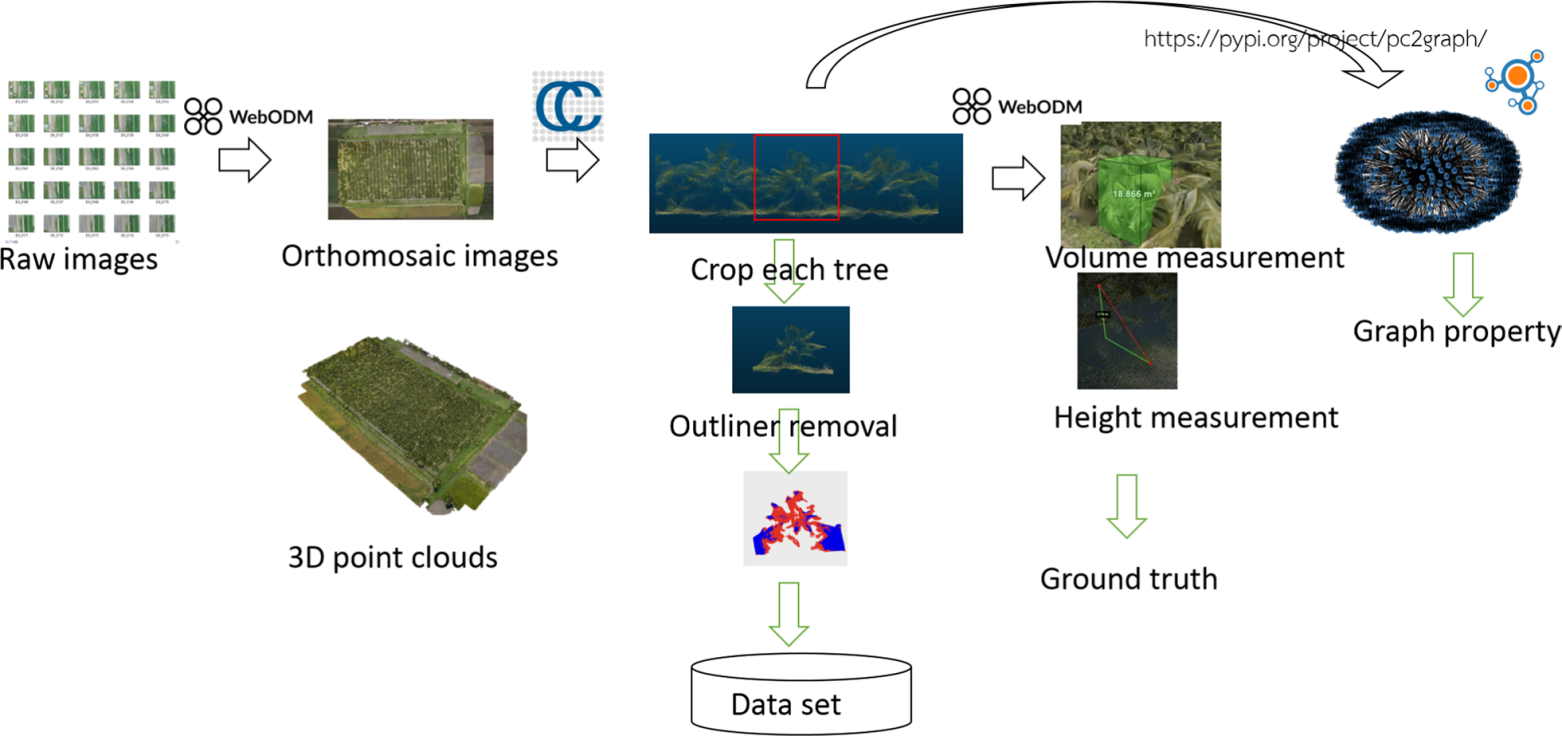
Processing methods

The open-source tools were applied for all the pipeline steps as shown in Fig. 1:

1. WebODM [9] is based on OpenDroneMap (ODM) [10] which has a scheduler to process various image processing tasks. An orothomosaic image and 3D point clouds were constructed.
2. CloudCompare [11] was utilized to extract each tree from the large 3D orthomosaic in 1).
3. Preprocessing such as outliner removal was done using CloudCompare and Open3D statistical outliner removal for each tree [12].
4. WebODM is utilized to record the necessary annotations such as tree height, and volume.
5. The 3D point clouds of each tree were used to create graph data based on voxels using Open3D with K-Nearest Neighbour algorithm [13].
6. Finally, Networkx [14] library was used for graph construction and property extraction.

### 3D Feature extractions

After extracting each tree, the main stage is to extract its features which are useful for analytic model creation. Open3D library was used to extract point cloud properties for each tree. The point cloud is visualized in Jupyter Notebook and the library extracts the point cloud including the number of points, volume size, point distance, number of mesh, etc.

Along with each crop tree, the ground truth of the tree such as volume size, and height were collected from WebODM for target labels. The results from the two tools enable the inference model constructions.

For example, to find the relationship between the actual tree height and the tree height from 3D point cloud geometry. The tree height can be directly extracted from the point cloud data. In WebODM, there is a measurement tool that can measure the height of the tree in 3D space. In step 2 of Section 2.1, the selected trees were measured their heights in meter units.

Next, the bounding box of the corresponding tree in pixels was collected using the Open3D function as a feature input. OrientedBoundingBox in Open3D [12] was utilized and the bounding box coordinates were recorded for each tree.

### Graph features

After Step 3 of Section 2.1, the derived point clouds were exported as (*x*, *y*, *z*) coordinates. In constructing a graph, the K-nearest neighbor algorithm computes the neighbor coordinates and derives the edges and distance. Large voxels may result in a large graph leading to high computation time. The voxel was downsampled to reduce the computation. The downsampling ratio used is 0.4 and the neighbor threshold was limited to 100. These values can be adjusted properly depending on the memory resource.

Next, Networkx library was utilized to extract graph features [14]. The feature includes the number of nodes, edges, triangles, cliques, clustering, connected components, etc.

## Results

A total of 40 palm tree and coconut tree point clouds were extracted. For each tree, 12 attributes were collected in Tables 1, 2. In the tables, rows“abb_vol” and “obb_vol” correspond to axis-aligned bounding box and oriented bounding box respectively. “avg_distance” is the average distance from nearest neighbors. “bpa_mesh”, “convex_hull” and “poison” are different kinds of mesh algorithms. Each of them implies a different number of points and triangles shown in the corresponding rows. The statistical features of point clouds are also shown in Tables 1, 2 respectively. It presents the standard deviation, mean, min, max, and 25%-75% quartiles.

**Fig. 2:**
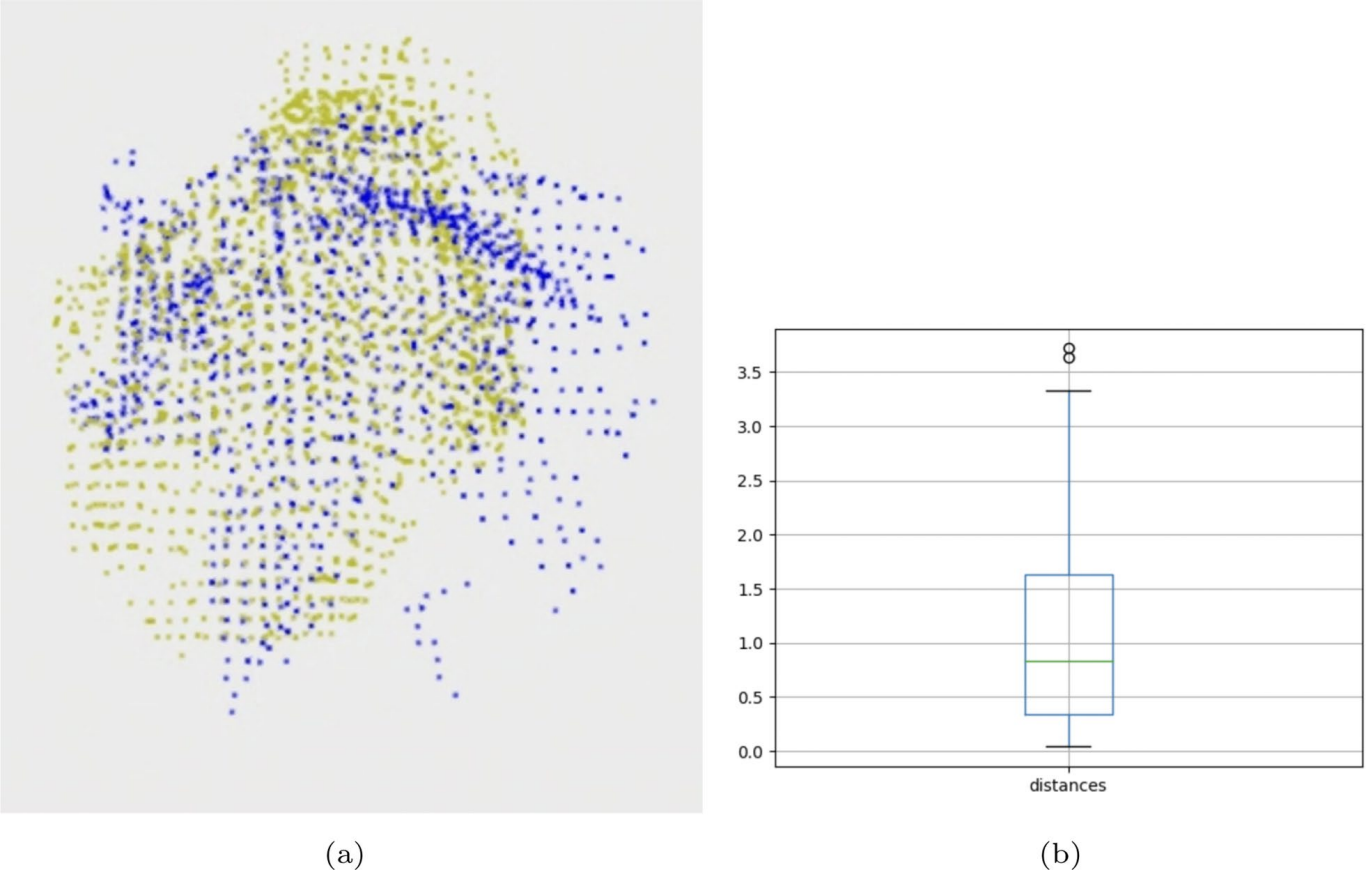
**a** Palm tree point clouds **b** box plot comparison

Fig. 2a visualizes the point cloud comparison between two palm trees (green points and blue points.) There are some differences between the width and height of the two trees as in Fig. 2b.

**Fig. 3:**
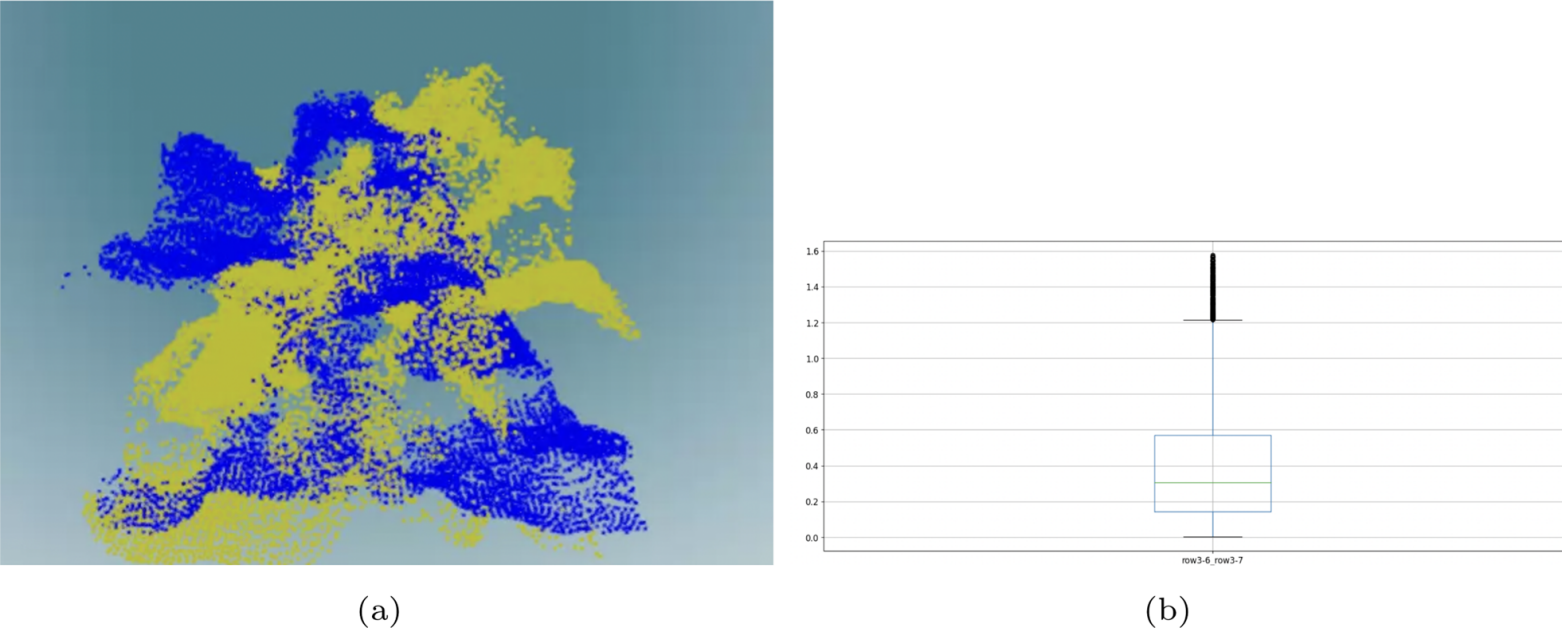
**a** Coconut tree point clouds **b** box plot comparison

Fig. 3a visualizes the point cloud differences between two coconut trees (green points and blue points.) and Fig. 3b presents the box plot of the difference values. There are more differences than in Fig. 2a.

**Table 1:** Statistics for palm point clouds

Attributes	Mean	Std	Min	25%	50%	75%	Max
#points	1,432.95	401.89	761.00	1,175.50	1,408.50	1,600.00	2,825.00
Abb_vol	657.15	265.96	232.66	452.72	597.84	829.73	1,276.22
Obb_vol	635.99	265.97	218.74	449.98	585.32	776.99	1,456.47
Voxel_grid	2.00	–	2.00	2.00	2.00	2.00	2.00
Avg_dist	0.22	0.02	0.18	0.21	0.22	0.23	0.27
Poison_avg_density	4.61	0.19	4.01	4.52	4.67	4.74	4.99
bpa_mesh _points	1,665.00	–	1,665.00	1,665.00	1,665.00	1,665.00	1,665.00
bpa_mesh _triangle	1,474.00	–	1,474.00	1,474.00	1,474.00	1,474.00	1,474.00
poison_mesh _points	10,680.00	–	10,680.00	10,680.00	10,680.00	10,680.00	10,680.00
Poison_mesh _triangle	5,435.00	–	5,435.00	5,435.00	5,435.00	5,435.00	5,435.00
Convex_hull _points	100.00	–	100.00	100.00	100.00	100.00	100.00
Convex_hull _triangle	52.00	–	52.00	52.00	52.00	52.00	52.00

Attributes	Mean	Std	Min	0.25	0.50	0.75	Max
Edges	500.80	78.64	378.00	446.50	480.00	550.50	690.00
Nodes	891.53	134.82	684.00	808.00	859.50	976.25	1213.00
Clique	891.53	134.82	684.00	808.00	859.50	976.25	1213.00
Average_cluster	0.00	0.00	0.00	0.00	0.00	0.00	0.00
g_eff	0.00	0.00	0.00	0.00	0.00	0.00	0.00
Triangle	25.04	90.19	−205.56	−38.11	14.30	89.26	219.18
g_reach	0.00	0.00	0.00	0.00	0.00	0.00	0.01
Min_cycle_basis	0.05	0.22	0.00	0.00	0.00	0.00	1.00
Max_ind_set	458.28	69.21	353.00	412.50	441.00	502.75	624.00
Max_matching	404.60	60.25	305.00	367.25	393.00	441.50	543.00
Num_isolate	0.00	0.00	0.00	0.00	0.00	0.00	0.00
s_materic	772.23	140.48	556.00	657.75	751.50	875.50	1095.00
Tree_branching_weight	26.19	47.03	−77.54	−8.75	25.05	55.71	157.52
Closeness_vitality	25.04	90.19	−205.56	−38.11	14.30	89.26	219.18
Num_clique	1171.00	0.00	1171.00	1171.00	1171.00	1171.00	1171.00
Min_vertex_cover	809.20	120.50	610.00	734.50	786.00	883.00	1086.00
Dominating_set	457.00	67.75	349.00	414.25	439.50	498.75	618.00
Clustering	25.04	90.19	−205.56	−38.11	14.30	89.26	219.18
Assort_coeff	0.00	0.05	−0.08	−0.04	0.00	0.02	0.13
Pearson_coeff	0.00	0.05	−0.08	−0.04	0.00	0.02	0.13
Avg_neighbor_degree	0.02	0.10	−0.27	−0.04	0.02	0.10	0.21
Avg_degree_connectivity	2.28	0.31	2.00	2.00	2.00	2.50	3.00
Num_connected_component	390.78	56.79	292.00	355.00	383.00	426.75	523.00
Num_connected	390.78	56.79	292.00	355.00	383.00	426.75	523.00
Min_weight_matching	408.68	60.86	310.00	372.00	395.50	444.50	549.00

**Table 2:** Statistics for coconut point clouds

Attributes	Mean	Std	Min	25%	50%	75%	Max
#points	15,090.37	3,582.08	8,404.00	12,463.75	14,753.00	16,970.75	28,328.00
Abb_vol	128.12	46.98	63.56	99.31	121.36	148.44	425.04
Obb_vol	180.70	57.04	82.83	145.33	174.90	200.69	428.49
Voxel_grid	2.00	–	2.00	2.00	2.00	2.00	2.00
Avg_dist	0.05	0.00	0.04	0.04	0.05	0.05	0.05
Poison_avg_density	5.95	0.24	5.57	5.78	5.90	6.05	6.78
bpa_mesh _points	4,492.00	–	4,492.00	4,492.00	4,492.00	4,492.00	4,492.00
bpa_mesh _triangle	15,946.00	–	15,946.00	15,946.00	15,946.00	15,946.00	15,946.00
Poison_mesh _points	122,161.00	–	122,161.00	122,161.00	122,161.00	122,161.00	122,161.00
Poison_mesh _triangle	61,016.00	–	61,016.00	61,016.00	61,016.00	61,016.00	61,016.00
Convex_hull _points	234.00	–	234.00	234.00	234.00	234.00	234.00
Convex_hull _triangle	119.00	–	119.00	119.00	119.00	119.00	119.00

**Table 3:** Statistics for graph data of palm point clouds

Attributes	Mean	Std	Min	25%	50%	75%	Max
Edges	764.35	231.85	469.00	577.25	739.50	890.50	1557.00
Nodes	1357.13	381.35	844.00	1053.75	1298.50	1555.50	2665.00
Clique	1357.13	381.35	844.00	1053.75	1298.50	1555.50	2665.00
Triangle	−39.06	109.91	−243.06	−102.67	−50.63	36.79	252.69
g_reach	0.00	0.00	0.00	0.00	0.00	0.00	0.01
Min_cycle_basis	0.05	0.22	0.00	0.00	0.00	0.00	1.00
Max_ind_set	698.48	197.01	434.00	534.25	669.00	798.25	1360.00
Max_matching	615.25	163.96	388.00	486.00	591.00	692.00	1184.00
s_metric	1195.18	457.80	627.00	820.25	1125.00	1440.25	2741.00
Tree_branching_weight	−104.12	65.92	−274.65	−144.51	−104.78	−61.27	49.94
Closeness_vitality	−39.06	109.91	−243.06	−102.67	−50.63	36.79	252.69
Num_clique	1171.00	0.00	1171.00	1171.00	1171.00	1171.00	1171.00
Min_vertex_cover	1230.50	327.92	776.00	972.00	1182.00	1384.00	2368.00
Dominating_set	696.70	196.87	431.00	540.25	666.50	805.50	1370.00
Clustering	−39.06	109.91	−243.06	−102.67	−50.63	36.79	252.69
Assort_coeff	−0.01	0.03	−0.06	−0.03	−0.01	0.02	0.06
Pearson_coeff	−0.01	0.03	−0.06	−0.03	−0.01	0.02	0.06
Avg_neighbor_degree	−0.03	0.08	−0.16	−0.08	−0.04	0.03	0.13
Avg_degree_connectivity	2.41	0.33	2.00	2.00	2.50	2.50	3.00
Num_connected_component	592.83	150.18	369.00	480.00	578.00	664.50	1108.00
Num_connected	592.83	150.18	369.00	480.00	578.00	664.50	1108.00
Min_weight_matching	621.98	167.79	392.00	492.50	594.50	709.25	1206.00

Tables 3, 4 present statistical data for 22 graph attributes derived from our methods. The selected graph attributes were related to nodes, edges, and subgraph structures. For instance, “max_ind_set” is the size of the maximum independent set. “max_matching” is the subset of edges in which no node occurs more than once. “num_clique” is the number of cliques “vertex_cover”

**Table 4:** Statistics for graph data of coconut point clouds

Attributes	Mean	Std	Min	0.25	0.50	0.75	Max
edges	500.80	78.64	378.00	446.50	480.00	550.50	690.00
nodes	891.53	134.82	684.00	808.00	859.50	976.25	1213.00
clique	891.53	134.82	684.00	808.00	859.50	976.25	1213.00
triangle	25.04	90.19	–205.56	–38.11	14.30	89.26	219.18
g_reach	0.00	0.00	0.00	0.00	0.00	0.00	0.01
min_cycle_basis	0.05	0.22	0.00	0.00	0.00	0.00	1.00
max_ind_set	458.28	69.21	353.00	412.50	441.00	502.75	624.00
max_matching	404.60	60.25	305.00	367.25	393.00	441.50	543.00
num_isolate	0.00	0.00	0.00	0.00	0.00	0.00	0.00
s_materic	772.23	140.48	556.00	657.75	751.50	875.50	1095.00
tree_branching_weight	26.19	47.03	–77.54	–8.75	25.05	55.71	157.52
closeness_vitality	25.04	90.19	–205.56	–38.11	14.30	89.26	219.18
num_clique	1171.00	0.00	1171.00	1171.00	1171.00	1171.00	1171.00
min_vertex_cover	809.20	120.50	610.00	734.50	786.00	883.00	1086.00
dominating_set	457.00	67.75	349.00	414.25	439.50	498.75	618.00
clustering	25.04	90.19	–205.56	–38.11	14.30	89.26	219.18
assort_coeff	0.00	0.05	–0.08	–0.04	0.00	0.02	0.13
pearson_coeff	0.00	0.05	–0.08	–0.04	0.00	0.02	0.13
avg_neighbor_degree	0.02	0.10	–0.27	–0.04	0.02	0.10	0.21
avg_degree_connectivity	2.28	0.31	2.00	2.00	2.00	2.50	3.00
num_connected_component	390.78	56.79	292.00	355.00	383.00	426.75	523.00
num_connected	390.78	56.79	292.00	355.00	383.00	426.75	523.00
min_weight_matching	408.68	60.86	310.00	372.00	395.50	444.50	549.00

**Fig. 4:**
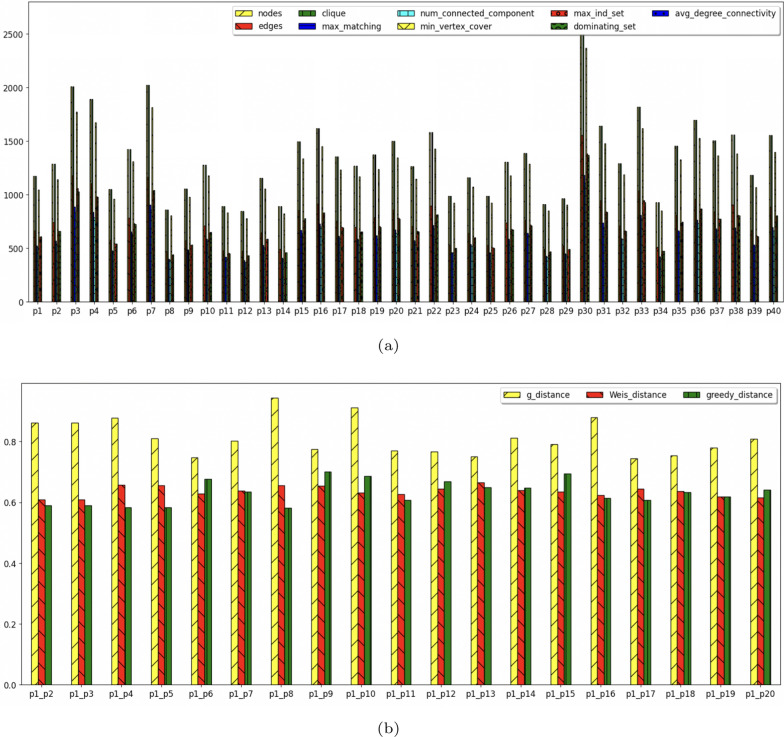
**a** Palm tree graph attributes **b** distance comparison

**Fig. 5:**
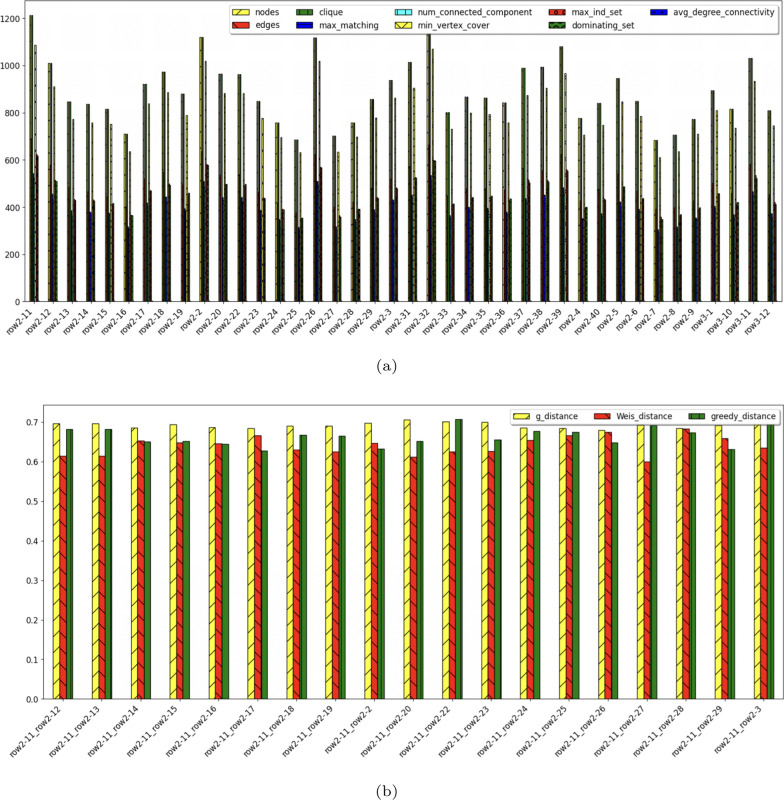
**a** Coconut graph attributes **b** distance comparison

Figs. 4a, 5a visualize the graph attributes of twenty palm trees and coconut trees respectively. Figs. 4b and 5b visualize the three types of distances of the two data sets.

## Discussion

For the 3D point clouds, the number of points of the coconut tree is more than that of the palm tree while the volume of the coconut tree is less than that of the palm tree. It may be noticed that the standard deviation of the coconut data set is more, implying data may not be cleaned enough.

One reason is the coconut tree is more difficult to crop since the shape of the tree top is quite varied. The ground point clouds attached to each tree during the cropping process overlap those of the tree which induces the outliers more than in those of the palm tree. Therefore, the coconut’s mesh size is larger than that of a palm tree. Nevertheless, the derived volume and density can be used to estimate the tree size and richness after normalization has been done.

When properly cleaned, the derived properties can be used to build a machine-learning model estimating the crop size. To expand the usage, the algorithm to segment each tree point cloud automatically can be derived and the volume estimation can be performed for each tree. This will reduce the manual measurement and increase the effectiveness of inspecting the crop size.

Comparing the two graph data sets, though we use the same parameter setting to produce the values, the palm tree point clouds seem to be larger than those of the coconut trees. For the palm tree, there are some large trees for example, p30, which can be seen by a large number of nodes and connected components, (Fig. 4a), and for the coconut tree, there are a few that have about the same size such as rows 2-11, row 2-20, rows 2-27, etc. as in Fig. 5a.

The values of all distances are close to each other for the coconut data set, while the difference is more for the palm data set between g_distance, Weis_distance, and greedy_distance.

**Fig. 6:**
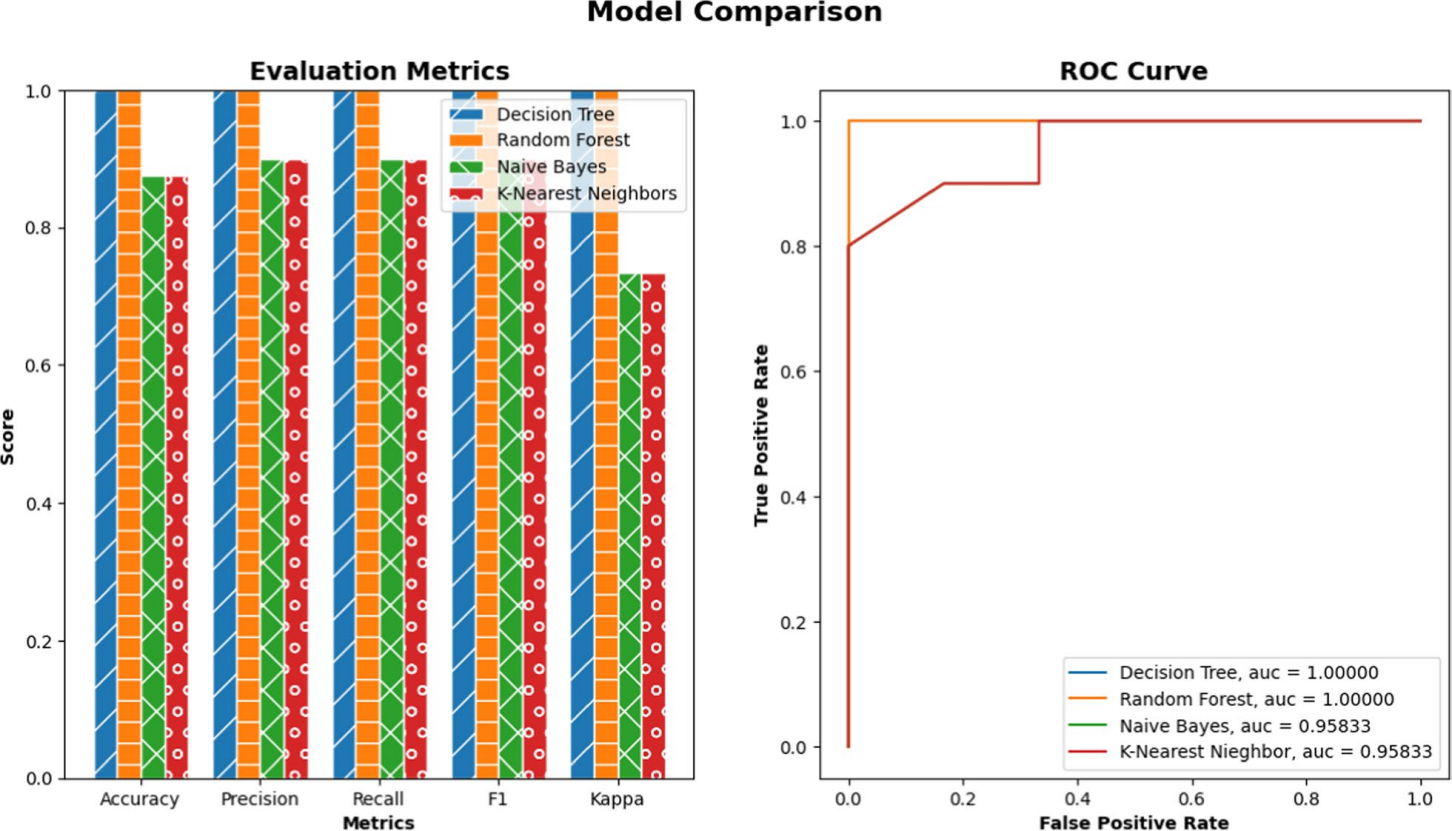
Comparing several classification

For the derived data sets, and graph attributes, we demonstrated the classification and clustering results considering two classes: coconut and palm. Fig. 6 presents the score of each classification method. All approaches can distinguish coconut from palm trees.

**Fig. 7:**
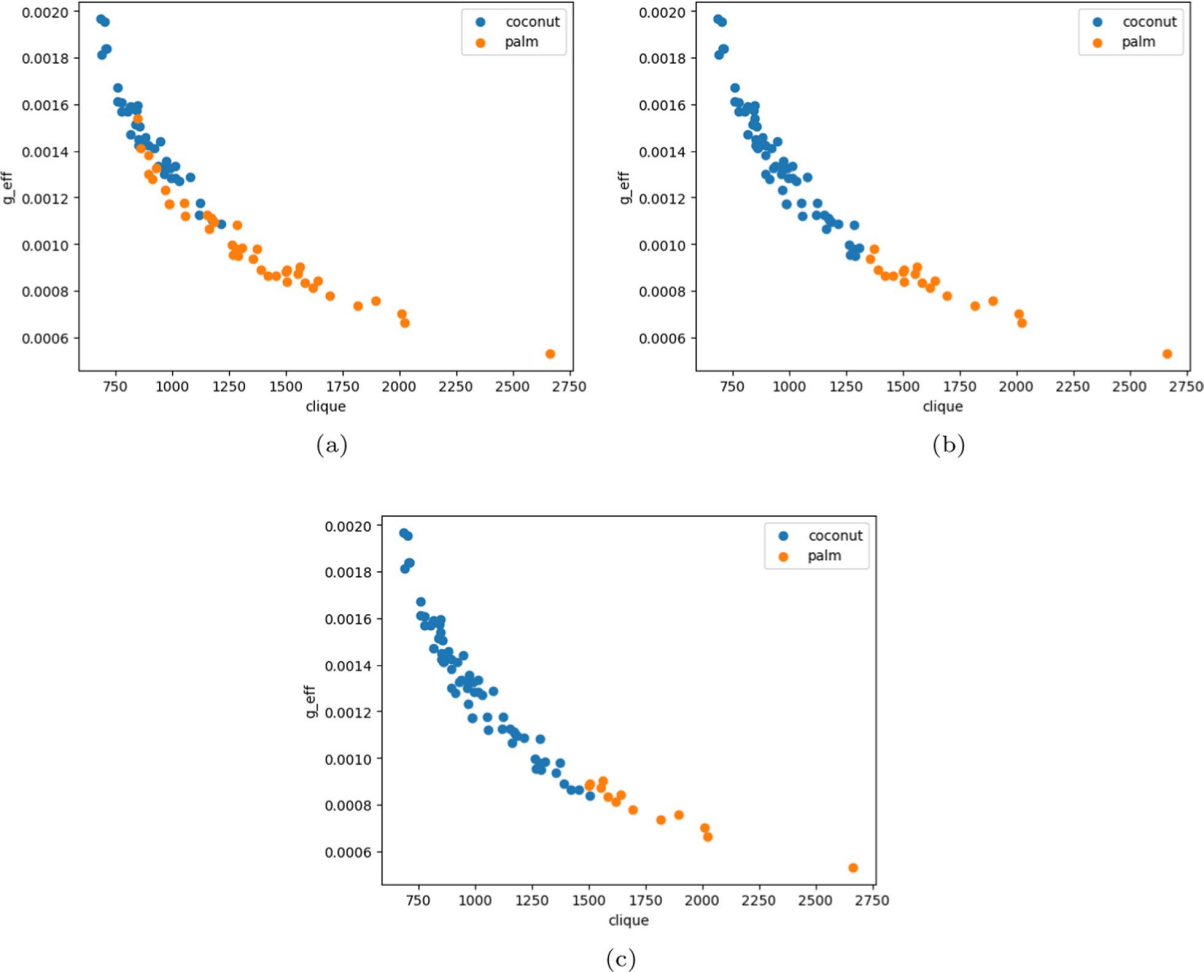
Comparing clustering approaches. **a** presents the original clusters while **b** shows the clusters obtained by K Means and **c** shows the clusters obtained by Birch

**Fig. 8:**
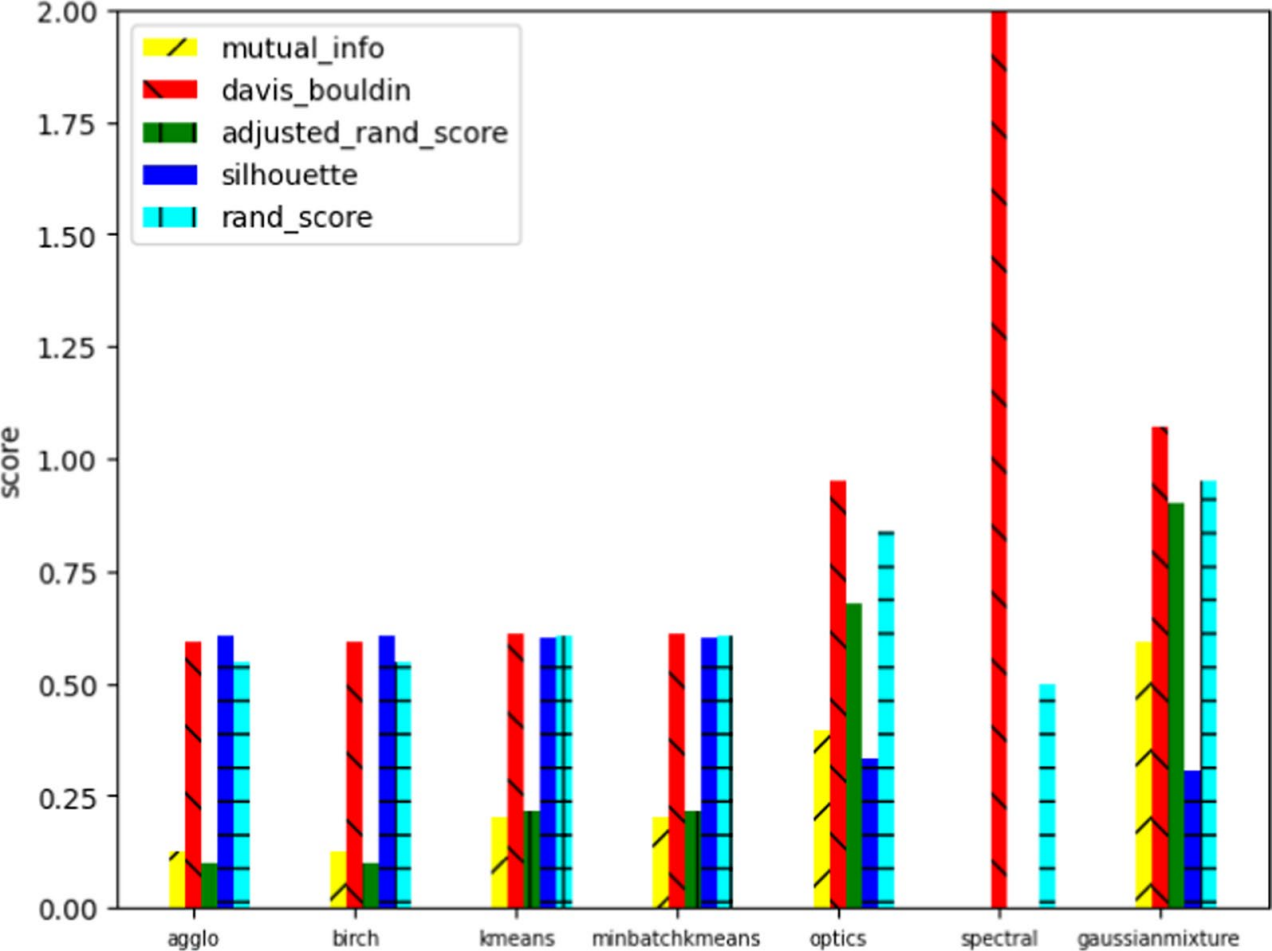
Scoring of several clusters

On the other hand, we combined both data sets and applied a clustering algorithm to cluster the data set. The purpose is to demonstrate the similarity of the two classes. Fig. 7 compares the results from two clustering approaches, K Means and Birch[15]. Two attributes ’g_eff’ and ’# clique’ are shown for the scatter plot. Compared to the original clustering in Fig. 7a, the K Means performs slightly better. Fig. 8 showed the common metrics for clustering results for five methods.

With these numeric attributes, other analytic opportunities are as follows.

1. The model to compute the size of the tree can be estimated by using these attributes.
2. Some attributes may infer the tree density, such as strongly connected components, average neighbor degrees, number of triangles, etc.
3. The inexact subgraph matching [16] can be applied to segment parts of the tree.
4. Graph neural network [17] can be applied to find the model to identify the substructure of the tree. The substructure may imply certain characteristics of the plant.

## Comparison to other works

Several works have been done about drone data sets. Most of them were found in urban survey areas. Drone mapper resources (https://dronemapper.com/sample_data/ provides some urban survey images from many places such as Colorado and Switzerland.

In agriculture, most published research utilizes data sets from orthomosaic images to perform analytics such as crop yields and 3D point cloud biomass. The whole orthomosaic image was used to calculate the yield indices and biomass. Commercial tools such as DroneDeploy, Pix4D, and Agisoft were utilized for preprocessing. Tunrayo et.al. [18] considered soybean grains yield prediction. Pixel4D was used to create orthomosaic for vegetation indices. Machine learning models were utilized for yield prediction. Acorsi et.al. [19] considered black oat trees with UAV images. Dronedeploy was utilized to perform the orthomosaic process and Agisoft Photoscan was utilized to create photogrammetry. They performed the biomass estimation for the derived photogrammetry. Worasit et.al. [20] considered forage crops and field peas. Vegetation indices and DSM were utilized to estimate biomass from point clouds. Most works provide processed or analyzed images.

Table 5 compared the previous works that take advantage of point cloud data sets in various ways. It is found that the most common data source for point cloud construction is 3D cameras. On the other hand, our work utilizes the 3D point clouds constructed from SFM (as in [21]) while we utilize WebODM and provide different applicability with graph features.

**Table 5:** Previous works that utilize tree point clouds

Work	Data set	Data source	Purpose	Data availability
Miao et.al. [22]	Maize	MVS img. acq. device	Labeling tools	Yes
Jiang [21]	Blueberry	SFM	Bush morphology	N/A
Wang et.al. [23]	Lettuce	3D camera	Segmentation	Yes
Morten et.al. [24]	Lettuce	3D camera	Weight estimation	Yes
Our work	Palm/ coconut	SFM	3D and graph features	Yes

## Limitation

The data set was first derived using WebODM. The point clouds for the whole field contain many trees of various sizes. To manually extract the tree, since the whole point clouds are large, a computer with powerful resources is needed. Moving in 3D space with CloudCompare can be slower if the computer memory is less than 16G. The alternative is to partition the whole point clouds into smaller ones and work on the partition.

This work focuses on individual trees, and future work includes the design of the algorithm to to automate the analysis, e.g., estimating the crop size for the whole field. The graph for the whole field must be generated and the subgraph segmentation using various methods can be applied [25–27].

## Data Availability

The data set is available at git@github.com:cchantra/3D-pointclouds.git. There are several folders. The folder “data” contains the point clouds of palm and coconut trees for 40 trees. The sample orthoimage obtained by OpenDroneMap is given in the google drive in README.md. The folder “csv” contains the data attributes obtained from for both point clouds and graph data. The code “process3d.ipynb” demonstrates the point cloud processing mentioned earlier. The python program “pcd_get_prop.py” computes point cloud properties and saves to CSV. The folder “distance” is the code to compute various distance types of graph data. The program “visual_csv.ipynb” performs the comparison between the two data sets using classification and clustering approaches. It combined the 40 items of each data set and performed the tasks. The folder “pc2graph” is the modified version (from https://github.com/mattbv/pc2graph.) making it compatible with updated libraries. The code “graphvisual_pcd.ipynb” converts point clouds to graph data and visualizes the data using Networkx. The dependency of running the code is Open3D, Networkx, pandas, etc. shown in “requirement.txt”. The dataset and the processing code are published at https://github.com/cchantra/3D-pointclouds.
